# Phenomena and Evidence of Serpent Poison

**Published:** 1886-10

**Authors:** G. Archie Stockwell

**Affiliations:** Port Huron, Mich.


### Art. XX.—PHENOMENA AND EVIDENCE OF SERPENT POISON.

BY G. ARCHIE STOCKWELL, M. D. F. Z. S.
*Port Huron, Mich.*

Great as is the scope of chemistry and the power of the microscope, there are products of Nature so exceedingly subtile and mysterious as to yet defy the most delicate and searching of tests, and to set at naught the most powerful objectives. Chief among these is the septic provision that secures to more than sixty species of serpents deadly weapons of offense and defense. It is practically, if not generically the same in all, is also common to at least one *saurian*, and exhibits modifications in none that are not with good reason, attributable to differences in habit and habitat, and influences of climate, season, size, age, development, and other ordinary phenomena of life. Drying under exposure in small scales, nearly colorless, soluble in water, partially so in glycerine and ether (the latter affording traces of fatty matter), not at all in alcohol, it manifests slight acid reaction, retains its properties for inconceivable periods, developes no special odor or taste, and is pronounced innocuous by the alimentary tract, in that it may be swallowed in any quantities and under all conditions, without affecting health or life; all the poison derived from half-a-dozen of the most venomous and virile reptiles is powerless to afflict an unfledged sparrow when poured into its open beak.

In daintiness of mechanism and service, the inoculatory apparatus of the venomous reptile transcends the more exquisite appliances of modern art. The fang teeth, typical canines, two or more in number, situate at either side of the anterior portion of the upper jaw, are independent of, and anterior to, the general dentition, which is exceeded by some five or six times in point of size and length. Lying just within the lips, rivalling the finest needles in delicate pointing, recurved, slender, and hard, they fold backward along the gums when the reptile is passive concealed by a reflection or fold of the fuccal membrane, offering no impediment to the passage of food or act of deglutition. Each is permeated in its longitudinal diameter by a capillary canal, that is but a continuation of a deep fissure at the base of the tooth where it is enfolded by the capsule of a duct communicating with the salivary apparatus, its apical outlet appearing as a minute oval fenestrum and barely traceable groove, upon its anterior or concave surface. As the tooth is falciform, and the canal direct and straight, this relation is easily understood. At the base of the fang extending from a point just beneath the nostril, back, two-thirds of the distance to the labial commissure, is found a gland, the analogue of the human *parotid*, that secretes a pure mucous saliva, but with which, oftentimes, is discovered mingled in varying proportions, a pale, straw-colored, semi-oleaginous fluid—the venom proper. The nerve and arterial supply of this gland, relatively is enormous, the former especially, and though derived through the *trigeminus*, posseses individual ganglionic relations, leading to the surmise that venom may be but a deterioration of salivary products proper under powerful nerve stimulus, rather than a secretion *sui-generis*, and this is in some measure borne out by the microscope, by its extraordinary septic properties, and by the fact its flow is powerfully stimulated either by fear or rage, and to some extent by hunger; two serpents killed under like conditions and relations of life, the one first alarmed, the other taken unawares, the former invariably reveals an undue supply of venom *within the gland*, the other little or none at all. Venom as secreted or manufactured by the gland, slowly distills away to find lodgment in the central portion of the excretory duct (*Stenonian*), that is here enlarged to form a receptacle or bulb, and from which it may be had of perfect purity in varying quantities, according to circumstances. This bulb, moreover, is surrounded by an aponeurotic capsule, the outermost and strongest layer of which is directly derived from the *masseter* muscle, through which both gland and bulb are compressed at will, emptying and refilling the latter almost synchronously.

The first flow commonly exhausts the poison fluid in the bulb; the second is less virulent in consequence of being largely dilluted with saliva; and few efforts are required to wholly exhaust the venom, when pure mucous in nocuous saliva alone results, and the creature is rendered impotent to harm. To this fortuitous circumstance may be ascribed the low rate of mortality in those bitten, as compared with the possibilities exhibited by pure venom; the virus undergoes frequent exhaustion in the effort to procure daily food.

Further examination of the mouth reveals other interesting peculiarities that, however, do not pertain to venomous tribes alone. The superior maxillary, palatine, molar, hyoid, and inferior maxillary bones, present a series of loose articulations, or more properly, perhaps, attachments, by means of elastic, expansive ligaments, whereby the oral aperture may be enormously distended and made to conform to the size and shape of the object to be received and swallowed. The teeth also, that adorn the arch of the mouth and the palate as well as the jaws, by reason of the multiple osseous sections * in which they are imbedded, are isolated and retractile in application and operation, precluding escape of prey once seized.

When the offensive or defensive is assumed by the reptile, the fangs are drawn downward in reverse plane until they point directly forward through the lips, the parotids under excitement swelling to twice or three times their natural size, producing the lateral bulging of the face so often described as flattening of the head, and all the muscles are rendered tense almost to rigidity, as the lower portion of the body is drawn into a spiral coil, upon which the remainder and the head pivots, and whereby is obtained the powerful darting movement that enables the creature to reach out in every direction to a distance of three-fifths, or four-fifths, its own length. As the head reaches the extreme limit of the spiral compatible with instantaneous recovery of position, or the point where the blow is expected to be struck, there is contemporaneous action of the compressor muscles that forces the venomous secretion violently through the apical outlets of the fangs and deep into the bottom of the wound; or, failing this the fluid is hurled to considerable distances. General Sir Arthur Conynghame relates of the Natal ombozi or spitting cobra (*naja*

* The roof of the mouth and the palate are alike made up of articulate sections instead of being one bone.

*haemachites*), that one thus blinded an officer of Her Majesty's XV Regt. of Foot, at a distance of *forty-five feet* as it lay writhing in mortal agony, and that the latter did not recover his eyesight for nearly a fortnight.

With the infliction of the stroke the fangs are reversed whereby the tissues at the bottom of the wound are lacerated as if to ensure fatality—a contrivance so ingenious in its devilishness it is little wonder the Semitic races accept the serpent as the type of Supreme evil, especially as their act is neither mechanical or obligatory. Some serpents also, as the *fer de lance*, *black mamba*, and *water moccassin*, actuated by the most vindictive motives, coil and cling about the part bitten, resisting all attempts for removal, as if anxious to instill the very last drop of venom which can be produced; and I was informed by a gentleman, resident of Texas, after being bitten by a large rattlesnake, that the creature "galloped away, going with an up-and-down motion as if thrilled with pleasure," and when a place of security had been attained beneath a large rock, "lifted its head waving to-and-fro with unmistakable manifestations of delight."

On the removal of a fang, until such a time as one of the rudimentary and supernumerary canines should be developed and pushed forward to supply its place, the gland is inoperative. The production of a new fang, however, requires but a few weeks *at most*, hence I recommend those who purchase serpents under the supposition they are harmless by reason of such removal, to exercise great care and watchfulness in handling. Twice I have myself suffered from such carelessness, and have learned first to depend upon personal ocular evidence, and to secure the desired condition in the serpent by either removing rudimentary fangs or by cauterizing the lower portion of the duct thoroughly by means of needle or wire heated nearly to whiteness, when, for experimental purposes the glands may be stimulated and the virus obtained by means of a needle-pointed syringe.

The physiological manifestations of serpent venom are exceeding swift, the toxic and pathological phenomena induced no less so. It is immediately taken up by the circulatory system and conveyed to the great nerve centres, whereby rapid paralysis of those organs that derive motive power from these sources supervenes. The inoculation induces intense burning pain in the part bitten accompanied by swelling and surface discoloration—spotted with livid blotches—that may ultimately extend to a greater portion of the body; and this is followed by nausea, retching, fainting, coldness and all the evidences of collapse, that is speedily induced if relief is not obtained. A greater or less disorganization of blood takes place, not by coagulating its fibrin as is often surmised, but by attenuating, dissolving, and altering the forms of those placques and red-corpuscles, whose integrity *is life*, causing them to adhere one to another, and to the walls of their conduits. It is by this stasis of capillary circulation that œdema is indeed along with the peculiar livid flush. It is unnecessary, however, that all, or even a majority of blood-discs be destroyed, since fatality usually supervenes long before such consummation can be realized. The capilliary circulation suffers mainly, for obvious reasons, while the very size and calibre of the heart cavities and trunk arteries secure to them comparative degrees of immunity.

Of the dissolved and disorganized condition of the red-corpuscles, ample evidence is afforded by the passive hæmorrhages that are so frequently the sequel to recovery from the more immediate effects of serpent venom. Bleeding may take place from any or all of the mucous-membranes—bowels and gums more especially—from ulcerated surfaces, old scars and cicatrices, etc., defing to the utmost all styptics and hæmostatics; and the microscope will reveal not the slightest trace or indication of fibrin, the essential principle of coagulation. In a case that came under the care of the late Doctor David Brainerd of Chicago, hæmorrhage of this character was derived from the gums, and he further assured me the exhalations from the patient's body and lungs were such as to develop serious illness in attendants, so marked was the sepris! Even in dead tissue, venom procures decomposition with most extraordinary swiftness.

The myotic failure of the heart, paralysis of muscles of respiration and arrest of pulmonary circulation, reflexedly through the *par-vagum* and *great-sympathetic*, are among the earliest of physiological and pathological phenomena. Breathing becomes retarded and laborious, the lungs no longer receive their proper supply of oxygen, the blood fails to be relieved of its carbon and is returned through the arteries, doubly gorging the already poisoned capillaries of the brain; and in this we find ample cause for the train of cerebral symptoms that, commencing with drowsiness, pass rapidly into stupor, and on to profound coma, terminating in death.

With the evidence before us, to look for an "antidote" or specific is the height of folly; such is but a lingering relic of that superstition that led the ancients to seek an universal panacea, and the follies of the middle ages to dabble after the "philosopher's stone." The fate of the poisoned rests solely upon existing physiological conditions and relations—personal idiosyncrasies, habits and conditions of life and health, and the ever-varying chapters of accidents including low vitality of the serpent and non-vitality of venom—or such as may be induced. The poison and its attendant train of sequalæ follow in too swift succession to be overtaken and counteracted, even through hypodermic administration!—Moreover, the point of inoculation has no reference to the period of toxic incubation. It is possible oftentimes, however, to so assist and support nature, to tide over the period of danger until the system may rid itself of the poison. Iodine, and iodic preparations are the nearest approaches to antidotes within the scope of mortal skill, inasmuch, if quickly forced into the circulation they retard and restrain the disorganization whereby the integrity of the red-blood discs is threatened and lost, are possessed of marked antiseptic properties, and favor the production of adhesive inflammation whereby absorption is checked through the effusion and coagulation of lymph in the part bitten. But, when it is sought to discover a remedy whereby the pristine form, functions and energies of destroyed corpuscle shall be restored—which alone fulfills the indications of an *antidote*—it presupposes supernal powers whereby man can not only preserve, but *create life anew!*

Aside from the possible and problematical value of blood transfusion, the means available in serpent poisoning are: (1) The prompt arrest of circulation in the part affected and local venesection and : (2) Enforced cardiac and pulmonary action until the emunctories shall have succeeded in eliminating the major portion of the virus. The rules laid down for the mechanical relief of opium and morphia narcosis, which present marked parallels, are of especial value, and in connection with cutaneous transpiration cannot be too highly extolled. The motto of Doctor Waring, late of H. M. E. I. service, "Enforced, continuous, violent, physical exercise, first, last and always until safety is assured !" fulfill every indication, for so long as cardaic and pulmonary action exists, death is impossible. The late Doctor Spillsbury, Surgeon General at Calcutta, relieved a favorite horse-keeper who had been bitten by a cobra (of such unusual size and virility that he was already looked upon as dead by his relatives) by fastening his hands to the back of a gig and forcing him, *willy, nilly* to keep up with the rapid motion of the horse—between nine and ten miles an hour—through a circuit of some thirty miles. In consequence, the man was rendered almost powerless through fatigue, and his body bathed by profuse perspiration, but suffered no further ill effects from the poison or the enforced exercise. The native population of India also, in spite of the popular opinion that they are familiar with antidotes for serpent venom, on the contrary are remarkably free from such superstitions, and that appears to be the especial prerogative and concomitant of advanced civilization ; this I can affirm from personal knowledge and evidence, and is corroborative of the assertion that " civilization retrogrades as fast as it progresses ! " The Hindoo who is so unfortunate as to encounter the cobra's fangs, is at once seized by his relatives and friends, and constant violent exercise enforced at point of stick, and severe, even cruel beatings administered. Jugglers, serpent charmers, and others who handle performing serpents, rarely deprive them of their fangs, the pecuniary success of their exhibitions depending usually upon the evidence afforded of vigor and virility on the part of their pets ; they rely solely upon deftness and manual dexterity, and the well-known placidity and even temperament of the reptile, which never strikes unless alarmed or irritated, yet each and every one, realizing full well the risks to which he subjects himself, carries beneath the girdle a sharp, broad-bladed knife, whereby amputation may be instantly performed. A native of Jubalpore that I knew, had thus lost successively his left little, index, and ring fingers, and finally the hand itself.

Alcoholic stimulants are of value in so far only as they act mechanically in sustaining a sinking heart and flagging respiration, since they in no way modify the venom; and in more violent cases, they are more than useless in that they tend to prevent and retard elimination. An intoxicated person, far from enjoying the immunity his condition is supposed to secure, succumbs, if anything, more rapidly than he who has full control of his senses. A few years since, in treating of a portion of this subject, I suggested, on theoretical grounds, the employment of *annyl-nitrite,* but after practical experience therewith I am prepared to assert its danger, since its effect as a motor excitant of cardiac action is strangely transitory, is suddenly lost, when paralysis follows almost instantaneously; that it tends to favor corpuscular disintegration, I am satisfied; *ethyl iodide* answers a better purpose, and may be administered both by inhalation and by the mouth.

It is an indisputable fact that there are individuals possessed of natural or acquired idiosyncrasies whereby they are enabled to resist the influences of the most virulent serpents and, perhaps, other specific and septic poisons. It will be remembered in the "Professor's Story" by Oliver Wendell Holmes, the woman living on "Rattlesnake Hill" brought a number of those reptiles from which this elevation derived its name, in her apron, to the Assistant Master of the "Institoot" with the remark "They never touches our folks!" So, too, Col. Matthews Taylor asserts his personal knowledge of a number of persons who regarded wounds inflicted by the cobra (*naja tripudans*) and tic paloonga (*daboii Russellii*) with all the indifference ordinarily manifested to mosquito and flea bites. Again, an itinerant vendor of nostrums offered for sale on the streets of the city of Melbourne, a compound claimed as an antidote to all serpent poisons, and evidenced its efficacy by causing himself to be repeatedly bitten by a number of reptiles exhibited. A Mr. Drummond, a magistrate of the city, convinced that the fellow was an impostor, and in order to convict him as such, (believing them to be naturally inoccuous, or else deprived of their fangs,) demanded a serpent should strike him, and becoming imperative, a small tiger snake (*heplocephalus curtus*) was handed him which inflicted a wound upon his wrist. He immediately exhibited all the evidences of serpent poisoning, and in spite of the application of the much vaunted remedy, and the exertions of medical men, within an hour he was a corpse. The vendor who was arrested, acknowledged the inefficacy of his nostrum, and acknowledged he, himself, enjoyed immunity from such poisons by reason of severe inoculation received in early life, further adding he knew "some people who were born so," who had put him up to this dodge as an easy mode of livelihood.

It is commonly surmised where such immunity is congenital that it is acquired *in utero* by the inoculation of the parent, and on this turns one of the points regarding the psychical conditions manifested by Elsie Venner, in the interesting tale of Doctor Holmes, already alluded to. Whether or no such inoculation may secure immunity to offspring is a mooted, not to say doubtful question, and viewed in the light of modern investigation would seem a sequel rather to some occult or accidental physiological phenomenon. The writer and certain of his relatives, for instance, are equally proof against the sepsis of *vaccinia* and *variola*, and in one instance at least it is known that the maternal parent suffered from small-pox during pregnancy; in the others it seems to be traceable to an epidemic of kine-pox that ravaged the neighborhood where they resided, at which time many persons exhibited painful ulcers upon the hands and arms that were ascribed to that bug-bear of the mothers of the last generation, especially during the vernal season—"humors in the blood!" It is well understood also that there are analogies between most septic poisons, as venom of serpents, and poisons of contagious fevers, inasmuch as recovery from either secures a degree of immunity from subsequent attacks of the same, perhaps for life; in other words, many animal as well as pathological poisons—such as *scarlitina, rubeola, variola, varicella, pertussis,* etc., have the power of so modifying the physical economy that it is incapable of affording lodgment and nourishment to *matines morbi*, whereby like toxic evidences are induced. A familiar example is found in the fact that prolonged residence in a noted mosquito region, leads to the attacks of these pests being passed over almost unnoticed, while they entail no little suffering to new arrivals, until they in turn have been thoroughly impregnated with mosquito venom.

In connection with the supposed correlation of serpent venom and the sepsis of certain fevers, it is interesting to note that the late Dr. De Humboldt, of Havana, in a paper read before the Royal Academy of Sciences, in that city, in 1852, not only assumed their identity, but advocated the inoculation of the former as prophylactic to the latter. He had inoculated numberless persons in Cuba and Mexico with exceedingly dilute venom, thereby securing far more favorable results regarding yellow fever than has ever accrued to the Pasteurization of *rabies*, but the fact that the experiments were not pushed to definite toxic manifestations, threw a discredit upon the whole that was subsequently confirmed by De Humboldt himself through careful segregated control investigation.

That the presence of one septic ferment is in greater or less degree inimical to others, is too well known to admit of argument or disputation, but the laws thereby are far from being accurately defined or fixed. So long as the sepsis of vacinea prevails, that of variola, or of itself recurrently, is impossible. In like manner Pasteur using a simple septic poison of whose precise nature he is ignorant, and of whose relations he is an unfit judge, by a series of illy conducted experiments upon animals, presumes he is able to wield a like power upon man, which he hopes to prolong indefinitely to personal ends and aggrandizement, the more so as he is presumably cognizant that the so called virus of *rabies* has no existence save in morbid imaginations, or as developed through other septic causes. The laws of impression and coincidence would seem to be his chief reliance, and those at all familiar with the latter, especially medical men who have given time and study to control experimentation, are well aware that coincidences are much more numerous, and prevail to a much greater degree than has ever been supposed or even imagined.

